# Metal Electrode-Free Halide Perovskite-Based Flexible Ultraviolet-C Photodetector with Large Area

**DOI:** 10.1186/s11671-022-03733-0

**Published:** 2022-09-21

**Authors:** Thi My Huyen Nguyen, Sean M. Garner, Chung Wung Bark

**Affiliations:** 1 Department of Electrical Engineering, Gachon University, 1342 Seongnam-daero, Sujeong-gu, Seongnam-si, Gyeonggi-do 13120 South Korea; 2 Corning Research and Development Corporation, One River Front Plaza, Corning, NY 14831 USA

**Keywords:** Flexible glass, Metal-free electrode, UVC photodetector, Halide perovskite

## Abstract

Ultraviolet-C (UVC) photodetector has appealed to a numerous number of research owing to its manifold applications in wireless communication, flame monitoring, and medicine. However, in addition to superior performance and high stability of recent studies, scalability and production cost are important factors for commercialization and practical implementation. In this study, a halide perovskite-based UVC photodetector was fabricated using spin-coating process and low-temperature annealing. Corning® Willow® Glass was selected as the substrate for the bottom-illuminated device due to its flexibility and exceptional optical transmission (approximately 60%) in the deep-UV region. The device had a vertical structure with a large active area (1 cm^2^) owing to the judicious utilization of electrodes. Under bent state with a curvature radius of 25 mm, the as-fabricated device exhibited high response and repeatability with an on/off ratio of 9.57 × 10^3^, a fast response speed of 45/46 ms (rise/fall times) at zero bias under the illumination of a 254-nm UV lamp. The results are based on a flexible and lightweight photodetector without the utilization of notable metal electrodes.

## Introduction

In recent years, lead halide perovskites have attracted significant attention in the field of optoelectronics due to their several advantages such as high carrier mobility, long exciton diffusion length, high absorption coefficient, low defect density, and tunable optical bandgap [1–4]. Additionally, its wide absorption range ensures response in the range of near-infrared wavelength to X-ray region [5–7]. Broadband photodetectors based on halide perovskite can be used for multiple applications such as image sensing, wireless communication, environmental monitoring, and medicine [8–11].

Low-cost halide perovskites have been utilized for the fabrication of wearable and portable photodetectors on commercial flexible substrates owing to advantages such as low-temperature fabrication and solution processability. For example, Dingjun Wu and coauthors recently reported an MAPbI_3_ nanowire photodetector by using welding strategy with outstanding on/off photocurrent ratio of 2.8 × 10^4^ and high detectivity of 4.16 × 10^12^ Jones [12]. Another approach, by using 1-butyl-3-methylimidazolium tetrafluoroborate (BMIMBF4) as an additive in fabrication MAPbI_3_ nanowire, the perovskite-based photodetector, exhibited not only ultrasensitive performance (specific detectivity of 2.06 × 10^13^ Jones) under 473 nm laser illumination but also ultrahigh stability for more than 5000 h without any encapsulation [13]. Flexible broadband photodetectors can be fabricated on commercial polymer substrates such as polyethylene terephthalate (PET), polystyrene (PS), polyethylene naphthalate (PEN), dimethylsiloxane (PDMS), and polyimide (PI) for the development of compact and lightweight devices [14–16]. However, a major drawback of these polymer materials is their limited wavelength response in the ultraviolet (UV) range. Deep-UV radiation is limited to approximately 300 nm, and the high optical absorption of substrates severely affects the device performance. In contrast, flexible Corning® Willow® Glass has a higher optical transmission in the deep-UV region [17, 18]. Hence, it can be used as an alternative to conventional flexible substrates for the development of ultraviolet–infrared (UV–IR) broadband photodetectors based on illumination through the substrate. Therefore, in this study, a device based on illumination through the substrate is developed, which can utilize the enhanced optical transmission through the substrate.

Regarding device structure, the interest in self-powered and flexible feasibility are characteristics to be considered. Addressing them, vertically-stacked devices exhibit fast electron/hole injection due to the shortening of the pathway in comparison with that of devices with a lateral structure [19]. Based on a built-in potential, photogenerated carriers can separate without the application of an external power supply. Supported by spiro-OMeTAD simultaneously functioned as an electron-blocking and a hole-transfer layer, photogenerated carriers quickly moved to the opposite electrodes to produce an electric current. Moreover, Lai et al. recently demonstrated that construction of a perovskite-based photodetector in vertical structure enables a remarkably higher mechanical flexibility than one in lateral structure [20]. In addition to the device architecture, the conductive polymer thin films are expected to be flexible electrodes with high mechanical durability to bending deformation rather than the rigid oxides (e.g., indium tin oxide, fluorine-doped tin oxide) and metal (e.g., Au, Ag, Pt) electrodes, especially for large-active area device [21]. Additionally, photodetectors are fabricated using complex methods and expensive metal electrodes such as Au, Ag, and Pt that increase the cost of production and impede large-scale production. To solve this problem, a transparent conductive polymer called poly(3,4-ethylenedioxythiophene) polystyrene sulfonate (PEDOT:PSS) is used as the photodetector electrode. Therefore, a simple spin-coating method can be used throughout the deposition process. A high-quality PEDOT:PSS thin film from an aqueous solution (PH1000) is used as the bottom electrode to allow the penetration of deep-UV light. On the top surface, a thin film obtained from the dissolution of dry re-dispersible PEDOT:PSS in isopropanol is in direct contact with the underneath layer. Therefore, the device is metal-electrode-free, flexible, and has a large active area. The bottom-illuminated device exhibited high performance in the UVC region with the self-powered operation and bending ability. A large photocurrent of 4.76 µA was generated by the device using metal-free electrodes due to the large active area. These results demonstrate a low-cost fabrication method for the development of halide perovskite-based broadband photodetectors which can be used in various industrial and scientific fields.

## Experimental

### Preparation of the Top Electrode

PEDOT:PSS solution was prepared by dissolving PEDOT:PSS orgacon™ dry (Sigma-Aldrich) in isopropyl alcohol (IPA) at a concentration of 1.3 wt.%. The dispersed solution was treated within 2 h of the ultra-sonication under 40% amplitude and 10 s on/10 s off of time pulse.

### Preparation of UV Photodetector

A flexible perovskite-based UV photodetector was fabricated using a solution-based spin-coating process and annealing at low temperatures. First, a 100-μm-thick Willow Glass substrate (20 mm × 20 mm) was cleaned with acetone, deionized water, and ethanol in an ultrasonic bath for 10 min during each cycle. The substrate was dried using nitrogen gas. Subsequently, a conductive bottom electrode was deposited on the clean glass by spin-coating Clevios™ PH1000 aqueous solution. The bottom electrode was treated with methanol as reported in a previous study [22]. A UV absorber film of halide perovskite was applied to the PH1000 layer via a two-step method. In the first step, a 1.3 M PbI_2_ solution in DMF:DMSO (0.95:0.5) was spin-coated at 2000 rpm for 30 s and annealed at 70 °C for 1 min. In the second step, a mixed organic halide solution which consisted of 60 mg FAI/6 mg MABr/6 mg MACl in 1 mL IPA was spin-coated on PbI_2_ layer at 4000 rpm for 20 s (loading time of 20 s) and the specimen was heated on a hot plate at 150 °C for 15 min to form the perovskite layer. Subsequently, a hole transport layer which consisted of 85 mg spiro-OMeTAD dissolved in 1 mL chlorobenzene, 28.8 μL 4-tert-butyl pyridine, and 17.5 μL Li-TFSI (520 mg Li-TFSI in 1 mL acetonitrile) was spin-coated onto the perovskite layer at 4000 rpm for 30 s. Finally, the top electrode was prepared by spin-coating 100 μL PEDOT:PSS solution in IPA on the surface of the spiro-OMeTAD layer at 1000 rpm for 60 s and heated at 50 °C for 5 min. Two copper wires were attached to the device using adhesive tape to connect them with the external circuit. The active area of the device was 1 cm^2^. For the bending test, the device was closely attached to a piece of flat plastic to determine the curvature radius and bending angle.

### Device Characterization

The crystal structure of the perovskite on the flexible glass was identified using X-ray diffraction (XRD; Rigaku DMAX 2200, Japan) with Cu Kα radiation (*λ* = 1.506 Å) in the 2*θ* range of 10°–45° and a scan rate of 5° min^−1^. The transmittance of 100-μm-thick Willow Glass in the deep-UV region and the absorption of the perovskite film on the substrate were measured using an ultraviolet–visible spectroscopy system (UV–Vis; Agilent 8453, USA). The recombination of electron−hole pairs in the perovskite was determined using a steady-state photoluminescence spectrometer (PL; QuantaMaster TM 50 PTI, USA) in the wavelength range of 750–800 nm with light excitation at 520 nm. The top views of perovskite film, PEDOT:PSS film on perovskite, and the cross-sectional images of the device were captured using scanning electron microscopy (SEM; S-4700, Hitachi, Japan) and a focused ion beam SEM system (FEI Nova Nanolab 200, USA), respectively. The electrical conductivities of two electrodes were measured by using a four-point probe system (Advance Instrument Technology, CMT SR-2000N). The spectral response was measured using the analyzer equipment (Keithley 2400 SourceMeter, USA) with a UV lamp (VL6.LC, France) as the light source.

## Results and Discussion

**Fig. 1:**
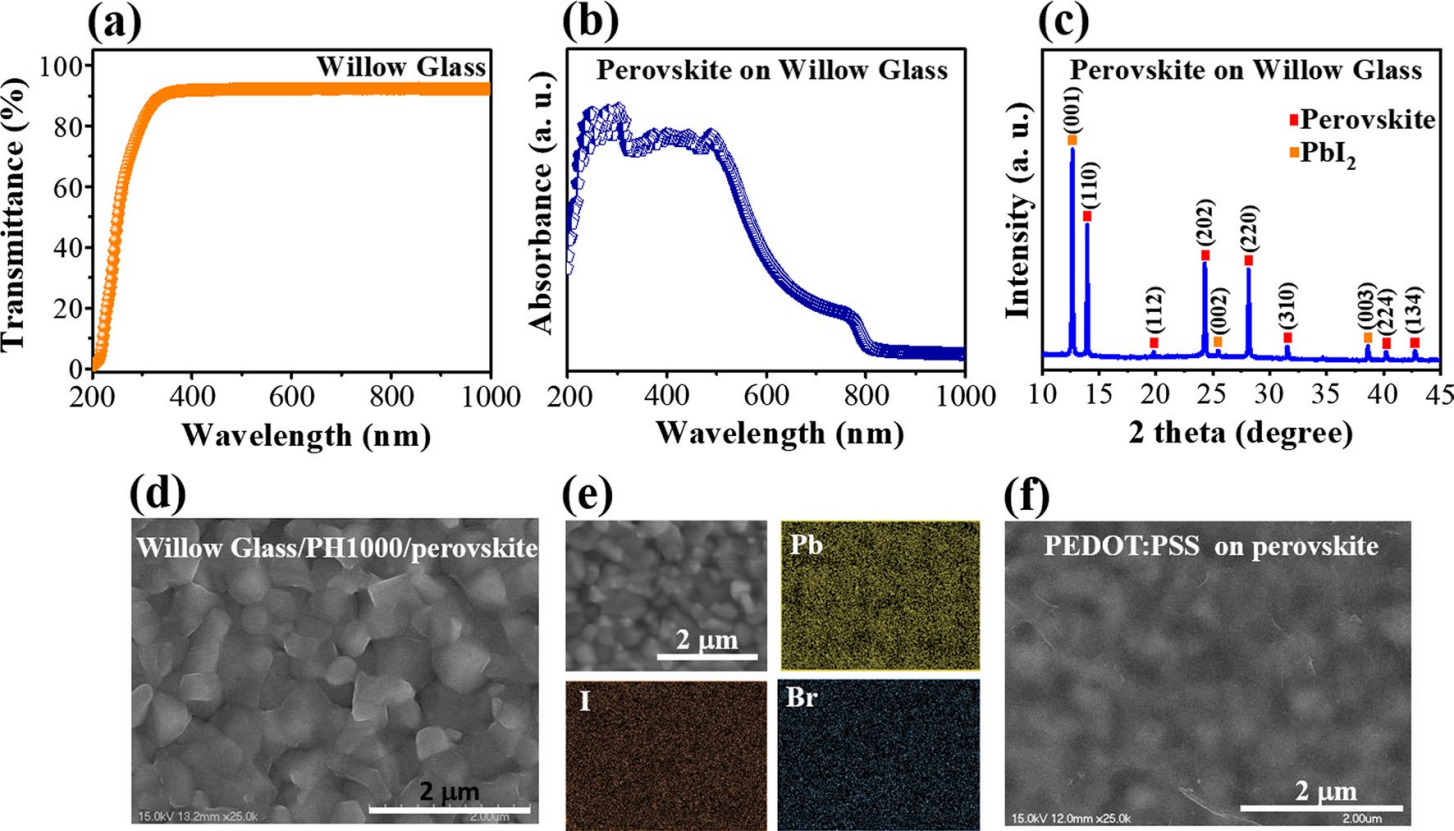
**a** Transmission spectrum of flexible Willow Glass substrate; **b** Absorbance spectrum and **c** XRD pattern of the halide perovskite layer on Willow Glass substrate; **d** SEM image and **e** Elemental mapping of the perovskite layer; and **f** SEM image of the PEDOT:PSS top electrode layer

The morphology, crystal structure, and optical properties of the halide perovskite-based UV photodetector are shown in Fig. 1. The transmission range of wavelengths in the range of 190–1000 nm for the 100-μm-thick Willow Glass is depicted in Fig. 1a. It can be observed that the transmittance was approximately 60% at a wavelength of 254 nm. Accordingly, UVC radiation in the range of 100–280 nm can pass through the flexible glass substrate to the active layer. The ultraviolet–near-infrared (UV–NIR) optical absorption of halide perovskite material on Willow Glass substrates is presented in Fig. 1b. The spectrum exhibits good absorption, particularly in the deep-UV region. The absorption of halide perovskite is consistent with the results obtained in previous studies [23–25]. Additionally, the XRD pattern of the absorbing layer (Fig. 1c) shows a set of prominent diffraction peaks at 2*θ*: 13.98, 19.81, 24.29, 28.19, 31.53, 40.26, and 42.8° corresponding to planes of (110), (112), (202), (220), (310), (224), and (134), respectively, that demonstrate the formation of the crystallographic phase of perovskite material on the Willow Glass [26–30]. The surface morphology of the active layer on flexible glass can be examined by the top-view SEM image and elemental mapping shown in Fig. 1d–e. A continuous and smooth film with large grains of perovskite crystals and a uniform distribution of Pb, I, and Br elements in the component layer can be observed in the figure. Furthermore, the surface of the top electrode can be clearly observed in the SEM image in Fig. 1f. It confirms a 100% surface coverage on the prior layer by the prepared PEDOT:PSS solution via a simple spin-coating method.

**Fig. 2:**
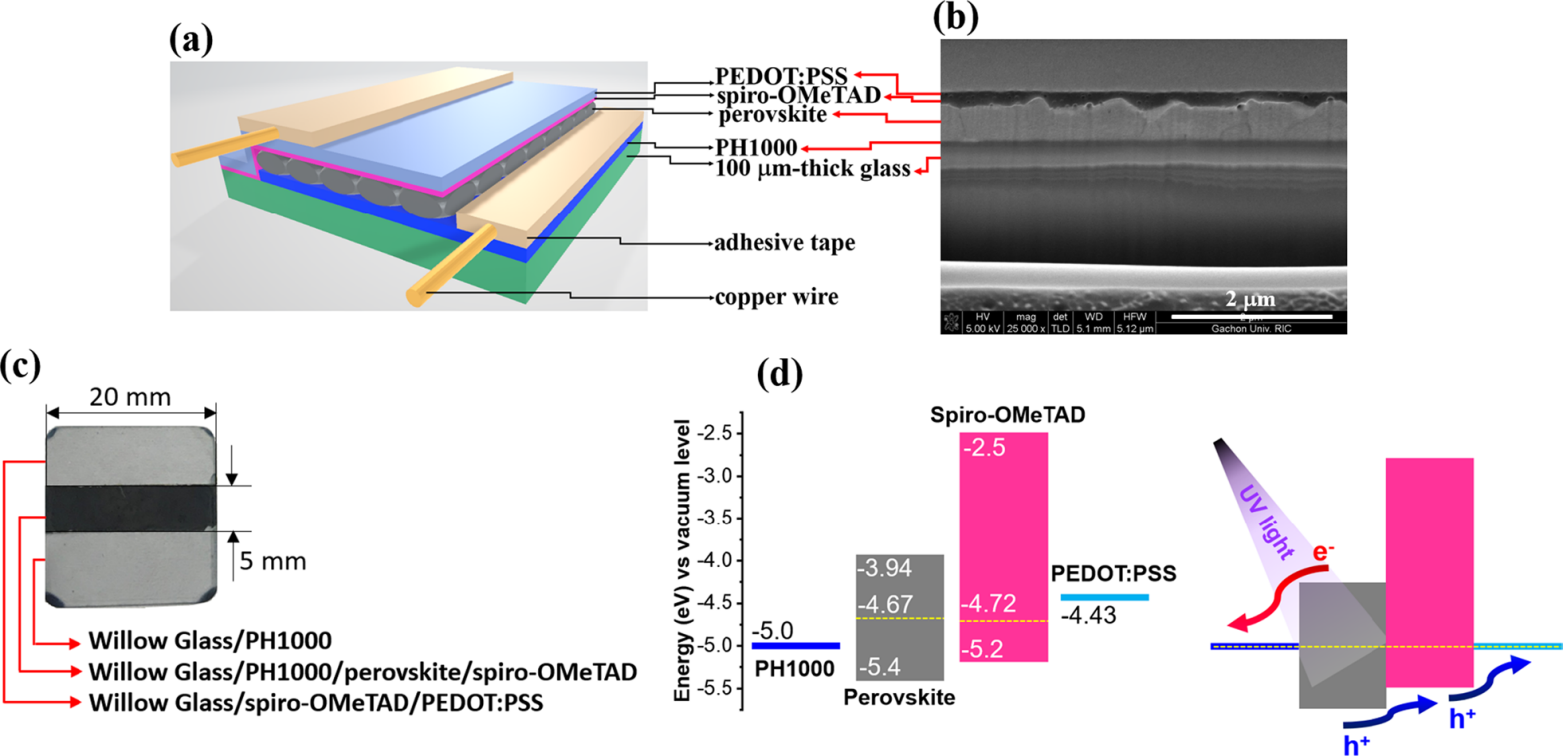
**a** Schematic diagram of the device; **b** Cross-sectional image of the device; **c** Photograph of the corresponding device; and **d** Energy diagram illustrating the charge carrier transport in the halide perovskite photodetector under UV light [31–34]

Figure 2a–c illustrates the schematic diagram, cross-sectional image, and a photograph of the photodetector device, respectively. It can be observed that the halide perovskite-based photodetector was constructed on a flexible Willow Glass with conductive polymer electrodes using two different types of solutions PH1000 aqueous solution (for the bottom electrode) and PEDOT:PSS slurry (for the top electrode). The layers of the components and the thicknesses can be observed in Fig. 2b. The thickness of the perovskite and PH1000 layers was  ~ 564 nm and ~ 82.5 nm, respectively. Furthermore, the layers of spiro-OMeTAD and PEDOT:PSS were complicated to distinguish because they have a good contact [35]. The top electrode layer can be determined due to the presence of a few defects that can be observed on a large scale. These defects were observed because PEDOT:PSS arrays were stacked on top of each other. The electrical conductivity values of PH1000 film and PEDOT:PSS film are 1032 and 223 S cm^−1^, respectively, suggesting efficient charge transport in the role of electrodes for the device. The energy diagram shown in Fig. 2c can be used to understand the mechanism of the photodetector fabricated on the flexible substrate. Upon the illumination of UV radiation, the energy penetrates through the transparent bottom electrode toward an active perovskite absorber layer. The electrons and holes are separated and self-driven to opposite sides. Owing to the built-in electric field and corresponding energy levels in the device, the photodetector can operate in the absence of a bias voltage. Furthermore, the two copper wires circulate the charge carriers through the external circuit to form current signals. Therefore, the self-powered photodetector can operate in a large active area without the requirement of thermal evaporation of noble metal electrodes such as Ag, Au, and a large amount of photocurrent was generated.

**Fig. 3:**
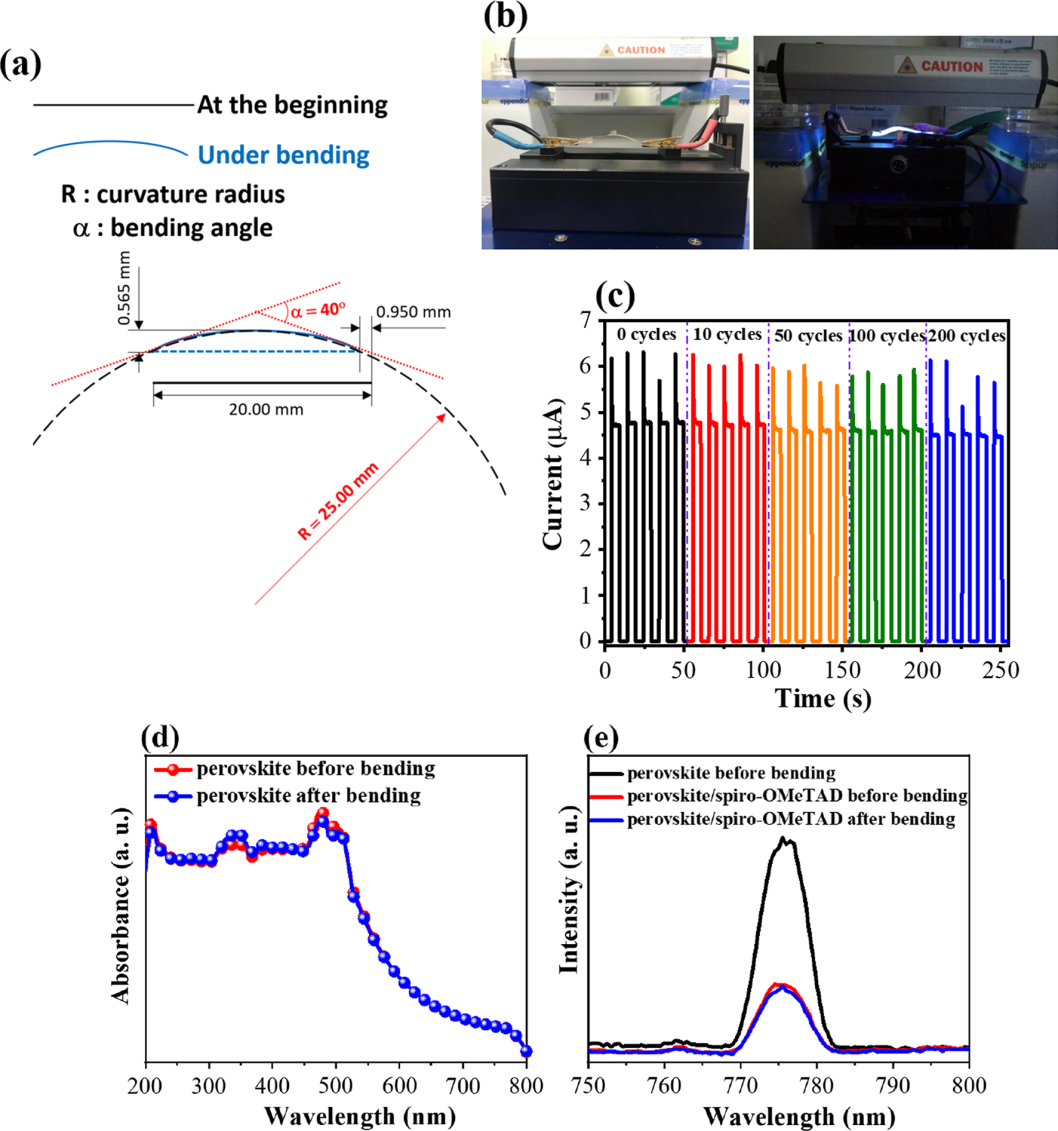
**a** Fitting of the bend test; **b** The device under test condition; **c** The performance of the photodetector after different bending cycles (0–200 cycles) at bending angle of 40°; **d** Absorbance spectra; and **e** Photoluminescence spectra of the perovskite films on Willow Glass substrate before and after the 200 bending cycles

The calculations and images shown in Fig. 3a and b, respectively, can be used to evaluate the performance of the photodetector on the flexible glass substrate during bending. The photodetector with a size of 20 mm × 20 mm was attached to a piece of plastic using tape. When the plastic was bent, the sample was bent as well. At the bending threshold (before breaking), the size of the sample under bending was measured to be 20 mm × 19.05 mm, and the height (distance between straight and bending) was measured to be 0.565 mm. Subsequently, a circular arc (black dash line) was fitted to overlap the sample arc under bending (blue dash arc). The bending angle and curvature radius were determined by drawing two tangents to the circle (red dash lines) and were equal to 40° and 25 mm, respectively. It can be observed in Fig. 3c that the photoresponse of the device exhibited stability and flexibility after repeatedly bent for 200 cycles at a fixed bending angle of 40°. This result demonstrated that the layers were not affected by the bending process at a moderate angle. The high mechanical durability was owing to the vertical construction of device [20]. Unlike the flexible polymer electrodes, the perovskite film can be cracked under tension during bending, which creates more surface defects to impede the transport of photogenerated charges toward electrodes in the horizontal direction. However, the negative influence of these defects on charge transfer will be suppressed because the charge carriers in the active area transfer to electrodes in the vertical direction. It is noted that thickness of the perovskite photoabsorber (~ 450 nm, Fig. 2b) is considered as the pathway of photogenerated charges in vertical direction. Indeed, the UV–Vis spectra of the perovskite layer on Willow Glass (Fig. 3d) measured at the beginning and after 200 bending cycles showed a similar absorption intensity, indicating maintenance of the optical property of the absorber material. Moreover, the PL measurements (Fig. 3e) were conducted to examine the effect of bending on carrier transport in the perovskite layer. In particular, the emission peak at 775 nm was assigned to the electron–hole recombination in the perovskite layer when it is under a light excitation of 520 nm. By using a hole transport layer of spiro-OMeTAD, photogenerated holes in the perovskite can move quickly to the spiro-OMeTAD side leading to reduce the band-to-band charge recombination, thus resulting in a significant reduction of PL peak intensity. Notably, the similarity of intensity was observed for devices after bending, indicating the unchanged charge transport in the as-prepared device before and after bending.

**Fig. 4:**
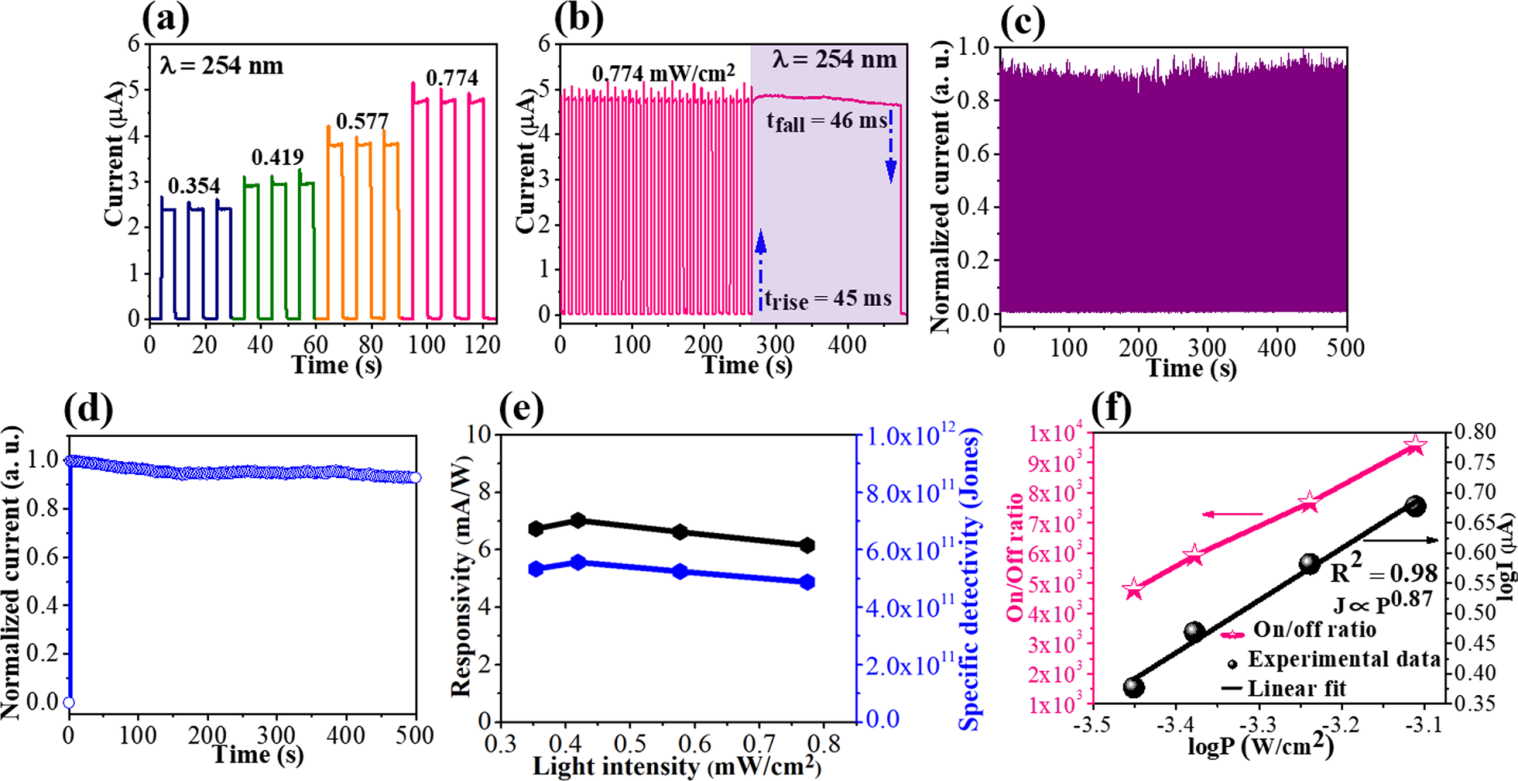
**a** Photoresponses of the detector with different incident light intensities from 0.354 to 0.774 mW/cm^2^ under bending test; **b** Transient photoresponse and stability of the detector under repeated on/off mode; **c** I-t curve with 500 on/off cycles; **d** Current with long-time UV exposure; **e** Responsivity and specific detectivity as a function of the power light; and **f** The logarithm of generated current under 254-nm irradiation and on/off ratio as a function of light intensity

The UVC response of the device at various incident light intensities was evaluated under the bent state. As depicted in Fig. 4a, the photocurrent generated increased monotonously with the power intensities from 0.354 to 0.774 mW/cm^2^ in the self-powered mode. The maximum value of photocurrent generated by the device was 4.76 μA, and it had a fast rise time of 45 ms and it returned to the initial dark current within 46 ms (Fig. 4b). Herein, the rise/fall time (t_rise_/t_fall_) is defined as the period from 10 to 90% of the peak value and from 90 to 10% of the peak value, respectively [23]. This response time as well as other critical parameters of our device are comparable with many reported flexible UV photodetectors in Table 1. Furthermore, Fig. 4c and d displays a reproducible and stable photoresponse of the device under multiple periodic on/off operations. In detail, the detector exhibited reproducibility during 500 consecutive on/off cycles accompanied by photostability under continuous 254 nm illumination up to 500 s. A quantitative assessment of self-powered photodetector was presented using two key parameters, responsivity (*R*) and specific detectivity (D^*^). They were expressed as *R* = (*I*_light_ – *I*_dark_)/*P*_opt_*S* and D^*^ = *R*/(2*qJ*_dark_)^1/2^, where *I*_light_ is the current under 254-nm UV illumination, and *I*_dark_ and *J*_dark_ are the dark current and dark current density, respectively. *P*_opt_ is the incident optical intensity, *q* is the electron charge, and *S* is the effective device area (1 cm^2^) [36, 37]. The values of *R* and D^*^ were calculated at different light intensities using the two above-mentioned equations and are plotted in Fig. 4e. A slight decrease in the curves can be observed with an increase in the power of light. The decrease was attributed to the recombination of defects present in the active area (1 cm^2^). This change can be verified by the power law: *I*_light_ $\propto$ *P*_opt_^θ^, where *θ* is the index of the power exponent. As shown in Fig. 4f, this exponent was determined to be 0.87 (*θ* < 1) for light intensity in the range of 0.354–0.774 mW/cm^2^, which demonstrates the dependence of photoresponse on incident light power caused by traps and defects [8, 38, 39]. Therefore, *R* and D^*^ values are proportional to ${P}_{\mathrm{opt}}^{-0.13}$ and have a tendency to decrease with high intensity. Similarly, the on/off ratio has a dependence on the power of light. Furthermore, it was observed that the magnitude of current generated under UVC illumination was thrice of the dark current at the investigated intensities. Additionally, the on/off ratio increased twice (4.79 × 10^3^–9.57 × 10^3^) due to the large active area. This indicated that the photodetector constructed on the Willow Glass substrate has a performance comparable with that of other devices in terms of high response, flexibility, and self-powered operation.

**Table 1:** Performance comparisons of previous flexible photodetectors

Device structure	Bias (V)	*λ* (*P*_opt_) [nm (mW/cm^−2^)]	*I*_photo_ (μA)	On/off ratio	Response time	Refs.
Willow Glass/PH1000/perovskite/ spiro-OMeTAD/PEDOT:PSS	0	254 (0.774)	4.76	9.57 × 10^3^	45/46 ms	This work
PET/ITO/TiO_2_/PEDOT:PSS	0	254 (0.6)	0.22	220	64/106 ms	[40]
PET/Ag/(Ti core/TiO_2_ shell) wire/Ag	-1	365 (18)	1	1.8	13.9/10.23 s	[41]
Mica/AZO/NiO/β-Ga_2_O_3_/ITO	2	265 (0.375)	5	10^3^	80/570 ms	[42]
PET/ITO/SnO_2_/perovskite/ITO	3	388 (11)	0.013	234	< 0.4 s	[43]
Paper/Ag/Sr_2_Nb_3_O_10_/Ag	5	260 (0.46)	1.7	1.1 × 10^2^		[44]
PET/MoO_3_/Au	10	380 (200)	0.3318	3.68 × 10^2^	0.94 s	[45]
Hair/AZO/ZnO/PVK/PEDOT:PSS	-5	365 (2.47)	5.5 × 10^–3^	11	110/200 ms	[46]

## Conclusions

A high-performance perovskite-based UVC photodetector was fabricated on a flexible Willow Glass substrate without using expensive metal electrodes. Spin-coating process and low-temperature annealing were used to fabricate the conducting polymers using two types of solutions, PH1000 (for the bottom electrode) and PEDOT:PSS (for the top electrode), to obtain a large active area. The device generated a maximum current of 4.76 μA at zero bias, and it exhibited a fast response time (*t*_rise_/*t*_fall_ = 45/46 ms) and a good on/off ratio under bent state. This study demonstrated a flexible photodetector that can operate under bottom-to-top illuminated UVC light due to high transmittance (60%) of the 100-μm-thick glass.

## Data Availability

All data supporting the conclusions of this article are included within the article.

## Abbreviations

PEDOTPSS: Poly(3,4-ethylenedioxythiophene) polystyrene sulfonate
XRD: X-ray diffraction
UV–Vis: Ultraviolet–visible
SEM: Scanning electron microscope
R: Responsivity
D*: Specific detectivity
PET: Polyethylene terephthalate
PS: Polystyrene
PEN: Polyethylene naphthalate
PDMS: Dimethylsiloxane
PI: Polyimide
